# Increased oxidative stress in asymptomatic current chronic smokers and GOLD stage 0 COPD

**DOI:** 10.1186/1465-9921-7-69

**Published:** 2006-04-28

**Authors:** Paula Rytilä, Tiina Rehn, Helen Ilumets, Annamari Rouhos, Anssi Sovijärvi, Marjukka Myllärniemi, Vuokko L Kinnula

**Affiliations:** 1 Division of Allergology, Helsinki University Central Hospital, Helsinki, Finland; 2 Pulmonary Medicine, Helsinki University Central Hospital, Helsinki, Finland; 3 Clinical Physiology, Helsinki University Central Hospital, Helsinki, Finland; 4 Department of Medicine, University of Helsinki, Helsinki, Finland

## Abstract

**Background:**

Chronic obstructive pulmonary disease (COPD) is associated with increased oxidative and nitrosative stress. The aim of our study was to assess the importance of these factors in the airways of healthy smokers and symptomatic smokers without airway obstruction, i.e. individuals with GOLD stage 0 COPD.

**Methods:**

Exhaled NO (FENO) and induced sputum samples were collected from 22 current smokers (13 healthy smokers without any respiratory symptoms and 9 with symptoms i.e. stage 0 COPD) and 22 healthy age-matched non-smokers (11 never smokers and 11 ex-smokers). Sputum cell differential counts, and expressions of inducible nitric oxide synthase (iNOS), myeloperoxidase (MPO), nitrotyrosine and 4-hydroxy-2-nonenal (4-HNE) were analysed from cytospins by immunocytochemistry. Eosinophil cationic protein (ECP) and lactoferrin were measured from sputum supernatants by ELISA.

**Results:**

FENO was significantly decreased in smokers, mean (SD) 11.0 (6.7) ppb, compared to non-smokers, 22.9 (10.0), p < 0.0001. Induced sputum showed increased levels of neutrophils (p = 0.01) and elevated numbers of iNOS (p = 0.004), MPO (p = 0.003), nitrotyrosine (p = 0.003), and 4-HNE (p = 0.03) positive cells in smokers when compared to non-smokers. Sputum lactoferrin levels were also higher in smokers than in non-smokers (p = 0.02). Furthermore, we noted four negative correlations between FENO and 1) total neutrophils (r = -0.367, p = 0.02), 2) positive cells for iNOS (r = -0.503, p = 0.005), 3) MPO (r = -0.547, p = 0.008), and 4) nitrotyrosine (r = -0.424, p = 0.03). However, no major differences were found between never smokers and ex-smokers or between healthy smokers and stage 0 COPD patients.

**Conclusion:**

Our results clearly indicate that several markers of oxidative/nitrosative stress are increased in current cigarette smokers compared to non-smokers and no major differences can be observed in these biomarkers between non-symptomatic smokers and subjects with GOLD stage 0 COPD.

## Introduction

The most important factor causing chronic obstructive pulmonary disease (COPD) is cigarette smoking which causes increased oxidative and nitrosative stress in this disease [1-3]. One major contributor to the increased oxidant burden in COPD is evidently nitric oxide (NO) since cigarette smoke contains the highest levels of NO to which humans are directly exposed [3]. Inducible nitric oxide synthase (iNOS), enzyme that produces the highest levels of NO in human cells and tissues, is also significantly induced by many of the mediators present in airway inflammation [1]. Markers of oxidative/nitrosative stress have been detected in the sputum and lung specimens of COPD [4-8]., but it is still unclear to what extent these markers can differentiate healthy smokers from non-smokers or smokers with symptoms but normal lung function parameters (FEV/FVC>70) from non-symptomatic smokers.

One of the most widely investigated non-invasive markers of nitrosative stress and airway inflammation is fractional exhaled NO (FENO). It is a sensitive and specific marker for eosinophilic inflammation in non-smokers [9], but its significance in smokers and its association with other markers of oxidative/nitrosative stress in the lung are poorly understood. FENO is significantly decreased in chronic smokers while it is variable in COPD [10-14]. There is evidence that FENO is higher in ex-smokers with COPD than in healthy non-smokers or current smokers with COPD [14], higher in COPD than in smokers with chronic bronchitis [15] and higher in COPD patients with reversible airflow limitation than in those with no reversibility [16]. Recent studies have indicated that FENO may vary at different levels of the airways [17]. FENO can be hypothesized to correlate with the numbers of eosinophils also in smokers [9,16]., but its association with neutrophil/macrophage associated airway inflammation needs further investigations.

Oxidative/nitrosative stress in moderate-severe COPD and its exacerbation has been confirmed by measuring the level/activity of oxidant producing enzymes and via the several "foot prints" of reactive oxygen species/reactive nitrogen species (ROS/RNS) mediated markers e.g. nitrotyrosine, 4-hydroxy-2-nonenal (4-HNE), other markers of lipid peroxidation, protein carbonyls and markers of DNA damage [2,3,18,19]. The classification of COPD that was launched in 2001 included a new group of subjects, those that have symptoms but normal lung function parameters (FEV/FVC>70) (GOLD stage 0 COPD) [20]. It is, however, unclear whether chronic symptoms actually lead to subsequent airway obstruction [2,21,22]. It is also unknown whether these above mentioned markers of oxidative/nitrosative stress can differentiate asymptomatic healthy smokers from those who have stage 0 COPD.

Non-invasive methods such as exhaled air, exhaled breath condensate and induced sputum have been widely used in the indirect assessment of COPD and its progression [14,23]. Of these techniques, exhaled air and induced sputum are relatively well standardized while the exhaled breath condensate, though promising, is still under evaluation [24-27]. In the present study, FENO, the inflammatory profile of the induced sputum, inducible nitric oxide synthase (iNOS), myeloperoxidase (MPO), lactoferrin (marker of neutrophils), eosinophil cationic protein (ECP), nitrotyrosine and 4-HNE as markers of oxidative/nitrosative damage of cellular proteins/lipids were investigated in 44 subjects, non-smokers or smokers. Smokers were either totally symptom free or if symptomatic, they were classified to have GOLD stage 0 COPD i.e. they had normal lung function parameters [20]. The features that might lead to difficulties in the interpretation of the findings such as allergies and reversibility in the bronchodilation test were excluded. In further analyses, the levels of FENO were also correlated with the inflammatory profile of the airways, and markers of oxidative/nitrosative stress.

## Subjects and methods

Altogether 22 current smokers (13 healthy smokers without any respiratory symptoms and 9 GOLD stage 0 COPD patients with chronic symptoms (cough and sputum production), mean smoking history 41 pack years) were included. At least 12 hours had elapsed from the last cigarette. Symptoms were assessed with the St Georges Respiratory Questionnaire, each symptomatic subject had both cough and sputum production. Smokers had no airway obstruction (postbronchodilator FEV1/FVC > 70%) and no significant reversibility (less than 10% reversibility in FEV1 after 400 μg of inhaled salbutamol). The control group included 22 non-smokers (11 never smokers and 11 ex-smokers at least 20 years from quitting of smoking and less than 15 pack years) with no history of lung disease. All the subjects participating in the study were non-atopic with no history of allergy. None of the subjects had suffered from respiratory infection for at least 8 weeks. One smoker had been prescribed inhaled steroids and 2 were using a β_2 _agonist as a relief medication. All other subjects were unmedicated.

The Ethics Committee of Helsinki University Hospital approved the study. All subjects gave full, informed consent. The study was registered by the hospital .

### Lung function measurements

Flow-volume spirometry was performed with a flow-volume spirometer connected to a computer (Medikro PC Spirometry, Medikro Oy, Kuopio, Finland) and Finnish reference values were used [28]. The pulmonary diffusion capacity for carbon monoxide (DLCO) and static lung volumes were measured by single breath technique [28].

### NO measurements

Exhaled NO (FENO) was measured with a chemiluminescence analyser (Sievers Model 270B NOA, Sievers Instruments Boulder, CO,US) by using a PC and software developed for the purpose [29]. The measurements were performed according to ATS guidelines [30]. Expiratory airflow used was 50 ml/s and the subjects exhaled against a flow resistor (Hans Rudolph, Model No.7100R, 200 cmH2O/l/s). The mean value from a 3-second period from the end-exhaled NO plateau was recorded. At least three successive FENO measurements were performed and the mean value was used for analysis.

### Sputum induction and processing

A standard procedure of induction was conducted using 4.5% hypertonic saline given at 5-min intervals for a maximum of 20 min according to the guidelines of the European Respiratory Society's Task Force [23]. Sputum samples were processed immediately after induction. All sputum macroscopically free of salivary contamination was selected and treated with dithioerythritol (DTE, Sigma, Germany) and phosphate-buffered saline. The suspension was centrifuged, and the supernatant was stored at -80°C for later assay. The pellet was resuspended and the viabilities and total numbers of the cells per gram of processed sputum were calculated by the trypan blue exclusion test. The sum of the viable and dead cells were calculated to obtain the total number of the cells. Coded cytospins were prepared and stained using May-Grunwald-Giemsa (MGG) method to obtain cell differential counts. Additional cytospins were frozen in -20°C for further assays.

### Immunocytochemistry for sputum cells

Polyclonal antibodies were used with the following dilutions: iNOS (Santa Cruz Biotechnology, US) 1:200, MPO (LabVision Corp., Fremont, US) 1:250, nitrotyrosine (Upstate Lake Placid, NY, US) 1:100, and 4-HNE (Calbiochem, San Diego, US) 1:8000, respectively. The cytospin samples were treated with Ortho Permeafix (Ortho Diagnostic Systems Inc., UK) for 40 min at room temperature for fixation and permeabilisation (for iNOS and 4-HNE) or with formalin (for nitrotyrosine and MPO). The endogenous peroxidase activity was blocked by incubation for 20 min with 0.3% hydrogen peroxide in PBS at room temperature. Zymed Broad spectrum antibody (Zymed Laboratories Inc., South San Francisco, CA, USA) was used as the secondary antibody for all antibodies except MPO. For MPO, Dako rabbit secondary antibody was used (Dako Cytomation, Glostrup, Denmark). For immunostaining, Zymed ABC Histostain-Plus Kit (Zymed Laboratories Inc.) was used according to the manufacturer's protocol. Thereafter, the samples were stained with Mayer's haematoxylin, followed by washing with distilled water. The immunoreactivities were expressed as the percentages of positive cells (from 400 cells in every cytospin) and as the total number of positive cells in the specimen (which was based on the total cell count obtained by trypan blue).

### Sputum supernatant measurements

Concentrations (μg/l) in thawed sputum supernatants of eosinophil cationic protein (ECP) and lactoferrin were determined using commercially available immunoassay kits (Pharmacia Diagnostics AB, Uppsala, Sweden and Calbiochem).

### Statistical analysis

Normally distributed data is expressed as mean and standard deviation (SD) and non-normally distributed data as medians and ranges. All the statistical analyses were performed using the SPSS 10.0 software program (SPSS Inc., Chicago, IL, US). Data for individual variables between two groups was analysed by the Mann-Whitney *U-*test. Correlations between variables were sought using the Spearman rank correlation test. A p-value of <0.05 was considered significant.

## Results

The clinical characteristics of the subjects are shown in Table 1. If the group of non-smokers was divided to two subgroups, i.e. 11 never smokers and 11 ex smokers, none of these characteristics differed significantly. If the group of smokers was divided into two subgroups, i.e 13 healthy smokers without any respiratory symptoms and 9 stage 0 COPD patients with symptoms, healthy smokers were younger, mean (SD) 53 (5.7) years than stage 0 COPD 64 (4.9) years (p = 0.001), had smoked less, 30 (13.0) pack years than stage 0 COPD 55 (17.7) (p = 0.001). FEV1/FVC was in all subjects over 70%, but healthy smokers had better lung function i.e. post bronchodilator FEV1/FVC 81 (4.1) % than stage 0 COPD 76 (3.4) % (p = 0.04).

**Table 1:** Patient's characteristics

**Variable**	**Never-smokers**	**Ex-smokers**	**Healthy smokers**	**Stage 0 COPD**
Number	11	11	13	9

Age (yr)	59 (50–72)	56 (51–64)	53 (42–61)	64 (57–70)
Sex (F/M)	2/9	2/9	3/10	2/7
BMI	27 (23–32)	26 (21–31)	27 (17–35)	29 (26–35)
Post bronchodilator				
FVC (l) *	4.6 (3.7–5.7)	5.0 (4.7–5.5)	4.7 (2.1–6.7)	3.4 (2.8–4.0)
FVC (% predicted) *	98 (81–110)	102 (101–103)	95 (65–122)	82 (67–104)
FEV1 (l) *	3.6 (3.1–4.2)	4.4 (4.1–4.6)	3.8 (1.7–5.3)	2.6(2.1–3.0)
FEV1 (% predicted) *	97 (81–113)	111 (109–113)	96 (63–122)	77 (62–100)
FEV1/FVC	79 (75–85)	87 (83–89)	81 (73–87)	76(71–83)
MEF 50 (% predicted)	87 (67–117)	131 (107–174)	85 (38–112)	57 (35–77)
MEF 25 (% predicted)*	98 (33–149)	151 (124–194)	87 (45–162)	53 (16–96)
Diffusion capacity (%)^†^	94 (85–106)	98 (87–111)	86 (69–109)	80 (64–99)

Data is shown as mean (range), *p < 0.05, ^† ^p < 0.01 (between all groups, Kruskall-Wallis test).

**Figure 1:**
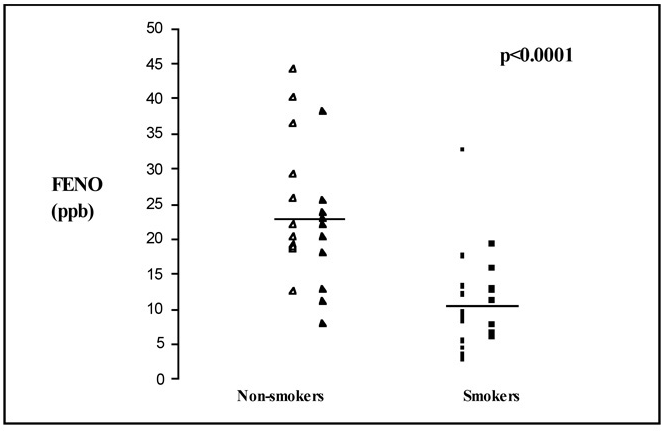
Exhaled nitric oxide (FENO) in the non-smokers (never smokers are presented with open triangles and ex smokers with filled triangles) and smokers (non-symptomatic are presented with open squares and GOLD stage 0 COPD with filled squares). There was a significant difference between non-smokers and smokers while the difference between the subgroups was not significant. Mean values are shown with horizontal bars.

FENO was significantly lower in smokers 11 (6.7) ppb compared to non-smokers 23 (10.0) (p < 0.0001) (Fig. 1.). If the two groups were analyzed separately, the level of FENO did not differ between these subgroups i.e. never smokers vs ex smokers (p = 0.193) or healthy smokers vs stage 0 COPD (p = 0.744). There was a significant negative correlation between FENO and BMI (r = - 0.320, p = 0.03).

**Figure 2:**
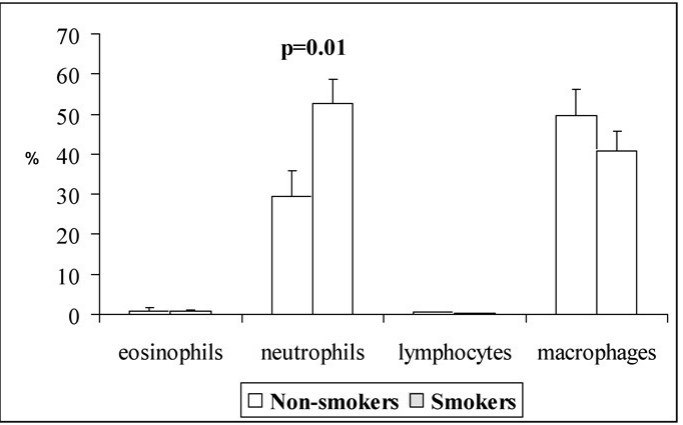
Inflammatory cell profiles in induced sputum of the non-smokers and smokers without symptoms or stage 0 COPD.

The cell profile of the induced specimens indicated elevated levels of neutrophils with no significant changes seen in macrophage or eosinophil numbers in smokers (Fig. 2.). If the groups were divided into the subgroups, the levels of neutrophils in these two subgroups were very similar. (never-smokers vs. ex -smokers neutrophils: p = 0.116, healthy smokers vs stage 0 COPD neutrophils: p = 0.23). The ECP level in the non-smokers was 26 (20) μg/ml whereas the level in smokers was 109 (245) μg/ml. However, the level of ECP did not differ between the two groups (p = 0.266) or between the subgroups significantly (p = 0.831 for non-smokers and p = 0.699 for the smokers).

**Figure 3:**
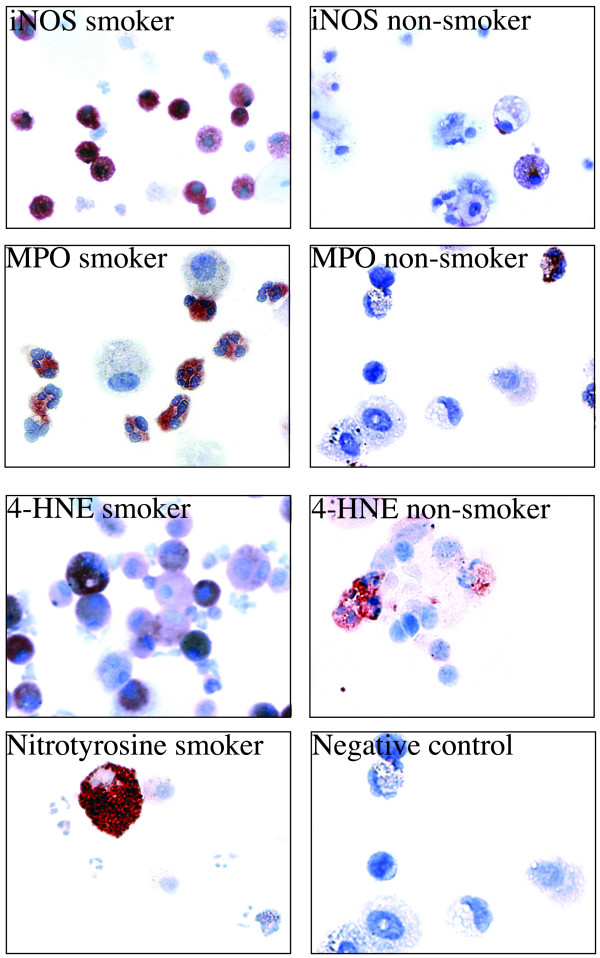
The expression of iNOS (a), MPO (b), 4-HNE (c) and nitrotyrosine (d) in the induced sputum of non-smokers and smokers. Negative controls showed no immunoreactivity.

**Figure 4:**
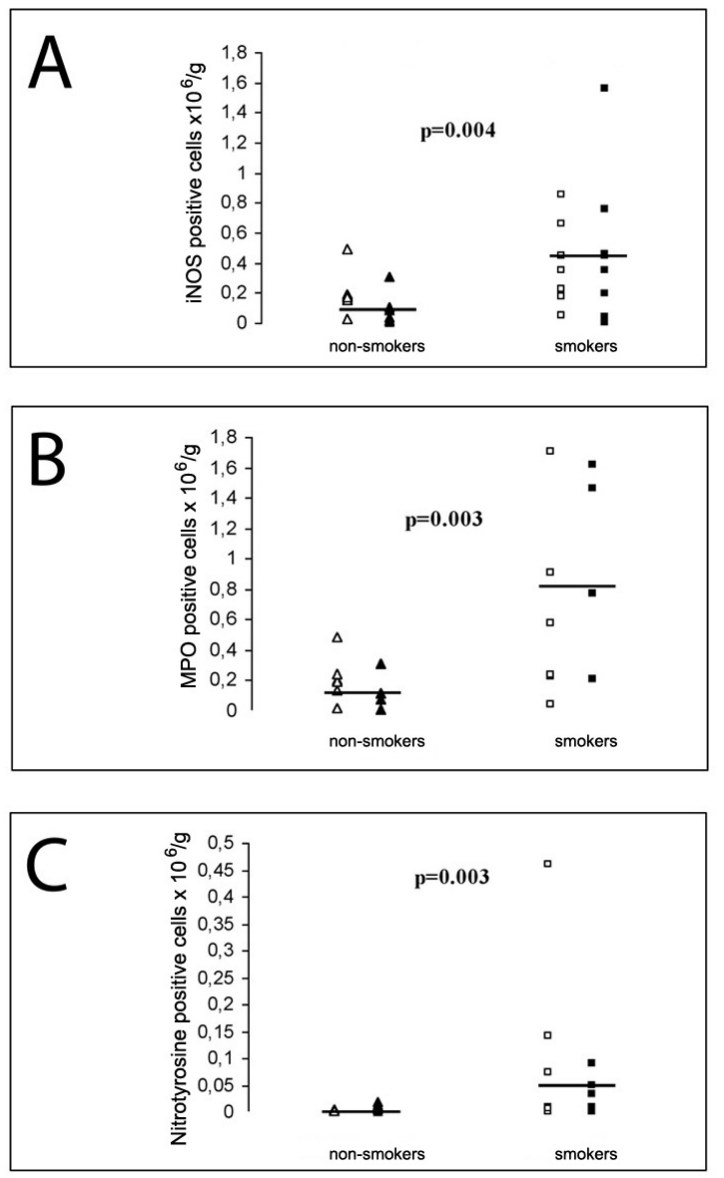
The number of iNOS (a), MPO (b) and nitrotyrosine (c) positive cells in the induced sputum of non-smokers and smokers. Mean values are shown with horizontal bars.

Induced sputum showed a marked positivity for iNOS, MPO, nitrotyrosine and 4-HNE in smokers as compared to non-smokers (Fig. 3). Both the percentage of the cells and the total number of the cells was calculated. The number of iNOS positive cells (Figure 4a) was higher in smokers than in non-smokers (p = 0.08, p = 0.004, respectively), the differences between the subgroups were: never smokers vs ex smokers p = 0.181 (%), p = 0.04 (total), non-symptomatic smokers vs stage 0 COPD p = 0.279 (%), p = 1.00 (total). The results for MPO were very similar (Figure 4b) smokers vs. non-smokers p = 0.01 (%), p = 0.003 (total), never smokers vs. ex smokers p = 0.536 (%), p = 0.09 (total), healthy smokers vs. stage 0 COPD p = 0.114 (%), p = 0.610 (total). Also for nitrotyrosine, the values between the subgroups were similar, smokers vs. non-smokers p = 0.01 (%), p = 0.003 (total), never smokers vs. ex smokers p = 0.898 (%), p = 0.414 (total) and healthy smokers vs. stage 0 COPD p = 0.689 (%), p = 1.0 (total), (Figure 4c). Representative samples (n = 4 for smokers and n = 4 for non-smokers) were also stained for 4-HNE, a marker of lipid peroxidation; also 4-HNE was mainly detected in the specimens from cigarette smokers and was higher in the smokers than non-smokers (p = 0.03, total). (Figure 3B). Among all these specimens, however, occasional cells from non-smokers also showed faint positivity.

Lactoferrin was analysed from sputum supernatants. Its levels were increased in smokers compared to non-smokers, 49.6 (27.8) vs 25.5 (11.2) μg/ml, p = 0.02. Again, there were no significant differences within the subgroups (p = 0.669 for never-smokers vs ex-smokers, and p = 0.17 for healthy smokers vs stage 0 COPD.

**Figure 5:**
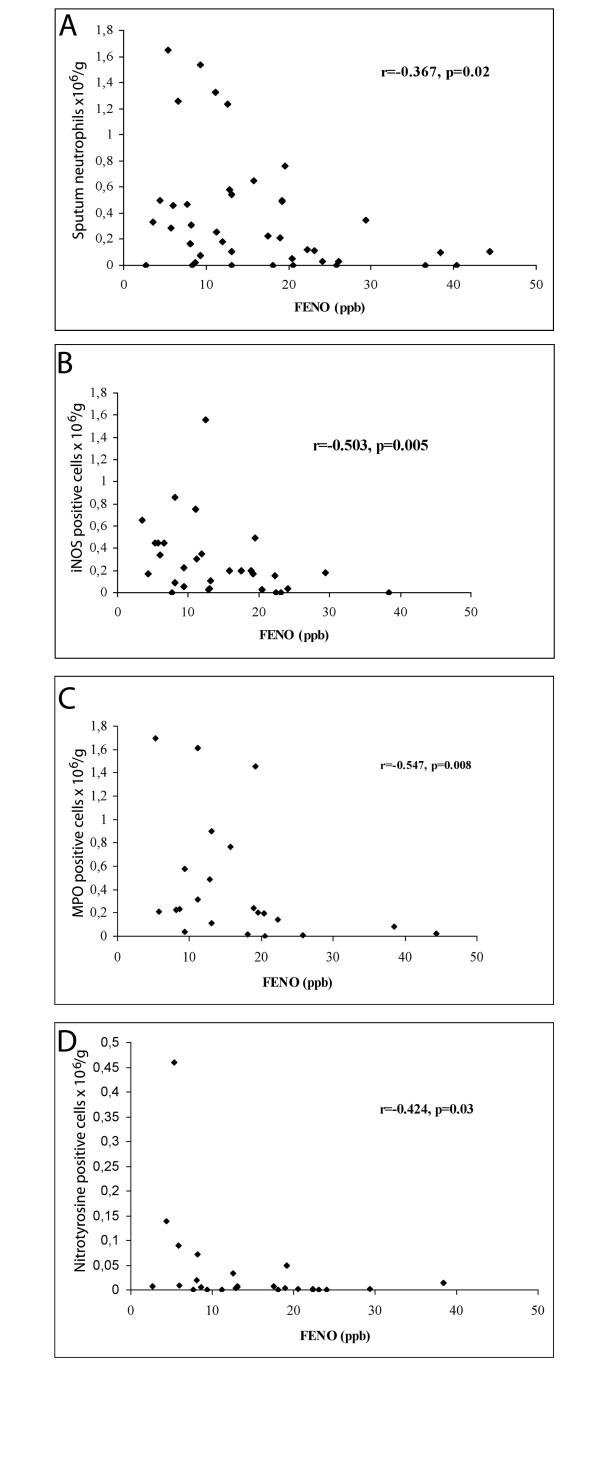
Association between FENO and sputum neutrophils (a), iNOS positive (b), MPO positive (c) and nitrotyrosine positive (d) cells in the induced sputum of non-smokers and smokers.

In subsequent studies, FENO was correlated with the markers of oxidative/nitrosative stress since the regulation of lowered FENO in cigarette smokers is poorly understood. These results indicated that there was a significant negative correlation between FENO and total number of neutrophils (r = -0.367, p = 0.02) (Figure 5a), FENO and total number of iNOS positive cells (r = -0.503, p = 0.005) (Figure 5b), FENO and total number of MPO positive cells (r = -0.547, p = 0.008) (Figure 5c) and FENO and total number of nitrotyrosine positive cells (r = -0.424, p = 0.03) (Figure 5d). Total iNOS and total nitrotyrosine had a positive correlation (r = 0.435, p = 0.038).

The correlation between these markers and small airway flow parameters MEF50 and MEF25 was also examined, but only total number of iNOS positive cells correlated negatively with MEF 50, r = -0.567, p = 0.006.

Given that atopy, asthma and reversibility are associated with increased FENO, special emphasis was placed not only on the patient histories but also on the sputum eosinophilia, FENO, and its association with eosinophils. The mean percentage of sputum eosinophils in the non-smokers was 1.0 (0.7–3.2) % and smokers 0.8 (0.3–1.3) %. There was a significant correlation between eosinophils and ECP (r = 0.661, p < 0.0001). There was a significant negative correlation between MEF 25 and sputum eosinophils (r = -0.374, p = 0.05) and a positive correlation between eosinophils and iNOS (r = 0.409, p = 0.02).

## Discussion

Our study reveals a significant elevation of iNOS, MPO, nitrotyrosine and 4-HNE positive cells in the induced sputum of chronic smokers with no airway limitation either without symptoms or with stage 0 COPD when compared to non-smokers. No major differences could be found in these biomarkers between the sputum specimens of healthy smokers and subjects with stage 0 COPD. These results also confirm the previous findings on decreased FENO in healthy cigarette smokers, but also indicate that decreased FENO is significantly associated with neutrophilic inflammation, MPO and elevated oxidant stress in the induced sputum specimens of these same individuals.

FENO is one of the most sensitive and specific markers of asthmatic/eosinophilic inflammation [9,31,32]. In contrast to asthma, FENO is highly variable in COPD, being decreased, unchanged or increased [7,10-14,33,34]. In general, elevated FENO has been found in COPD patients with reversible airway limitation and eosinophils [16]. As expected, FENO was decreased in smokers, but it did not differ significantly in the non-symptomatic smokers from those with stage 0 COPD. FENO significantly but inversely correlated with sputum neutrophils and MPO, but not with macrophages. This correlation is in contrast to that observed in patients with moderate/severe COPD [7] further highlighting the difficulties in the interpretation of FENO during the course of COPD progression. Cigarette smoke can cause elevated burden of NO and ROS both directly and by activation and recruitment of inflammatory cells and by activating oxidant/NO producing enzymes. NO rapidly reacts with and is consumed by the other ROS/RNS abundantly present in the airways. These reactions can lead to lowered FENO in cigarette smokers but also can explain the negative correlation between FENO and MPO as was observed in the present study. Also the negative correlation between FENO and iNOS might be the sum effect of NO produced by cigarette smoke and iNOS and these reactions between NO and ROS/RNS. It is, however, possible that the observed negative correlation with iNOS may also be related to the increased number of inflammatory cells as they express iNOS. Overall, our study suggests that the low FENO level in cigarette smokers also argues against asthmatic/eosinophilic inflammation.

Inducible NOS is increased in asthmatic airway inflammation, but can be decreased during corticosteroid therapy [8]. As with FENO, variable levels of iNOS have been reported in the lungs of COPD patients probably due to severity of the disease, smoking history, anti-inflammatory therapy or the various methods used for iNOS detection. INOS has also a cell specific expression in human lung, being located especially in the inflammatory cells but also in the bronchial epithelium [4,5,8,12,35,36]. In the present study, each subject had normal lung function parameters, at least 12 hours had elapsed from the last cigarette, and anti inflammatory therapy had been prescribed only to one smoker. The expression of iNOS was variable but in general the enzyme was more often expressed in smokers than in non-smokers but could also occasionally be detected in samples from non-smokers. The relative importance of iNOS activation in cigarette smokers as a NO producer and its involvement in COPD pathogenesis remains still unclear. Nevertheless, it may be likely that NO in smoker's airways is derived both from the cigarette smoke and endogenously from iNOS. Under these circumstances, the reactions betwen NO and ROS would lead to further production of other toxic metabolites such as peroxynitrite and these agents may cause nitration of proteins and lipids, theoretically even in smokers without airflow limitation.

Nitrotyrosine is a marker of oxidative/nitrosative stress that can be formed not only by iNOS activation but also by MPO [37]. The percentage of nitrotyrosine-immunopositive cells has been found to be higher in the induced sputum of COPD patients compared to non-smoking controls [4]. The patients in those previous studies, however, suffered from moderate/severe COPD This present study detected nitrotyrosine reactivity also in smokers without airflow limitation. 4-HNE, a marker of lipid peroxidation, has earlier been detected in a biopsy study of COPD patients [6]. In the present study, also 4-HNE showed clear positivity in the samples of cigarette smokers. Since not only cigarette smokers but also some samples of non-smokers expressed nitrotyrosine, our study suggests that this biomarker cannot be used as a reliable index of oxidant related lung injury or a predictor of COPD progression. Nitrotyrosine may, however, point to the presence of nitrated amino acids in the proteins/enzymes and this may have possible functional consequences such as enzyme inactivation.

Also MPO, a marker of neutrophil activation, is known to be associated with COPD, its exacerbation and decreased diffusion capacity [38-42]. but little is known about MPO in smokers with normal lung function parameters or mild COPD. In the present study, MPO could be detected more often in both groups of cigarette smokers with normal lung function parameters than in non-smokers. This is also in line with increased levels of lactoferrin in cigarette smokers, since lactoferrin is located in neutrophils. In agreement with the negative association of FENO with neutrophils, a corresponding negative association was also observed between the levels of FENO and MPO.

As far as we are aware, FENO has not been evaluated in different BMI groups, but it is possible that FENO may be related to the structure of the airways that may differ in various groups of the subjects and populations. In this study, FENO exhibited a significant correlation with BMI. These differences have not been included in the reference values of FENO but will need to be investigated more carefully in future investigations.

GOLD stage 0 COPD represents a group of symptomatic subjects who may be at risk of COPD developing although their lung function parameters (FEV1/FVC) are normal. Besides investigating to what extent smoking alone increases the levels of oxidant markers in the induced sputum specimens, another goal was to investigate if there are any differences in these biomakers between non-symptomatic smokers with normal lung function parameters and stage 0 COPD. We are not aware of studies where FENO or markers of oxidative stress such as MPO, iNOS or nitrotyrosine have been compared between these two groups. Never smokers, healthy smokers and symptomatic smokers have not been simultaneously included in previous investigations [4,7,8]. In the present study, several parameters of oxidative/nitrosative stress were generally increased in smokers when compared to non-smokers but were very similar in the two groups of smokers, both groups had long smoking histories. However, more studies, especially longitudinal ones will be needed to verify these findings with greater numbers of subjects and lung/sputum specimens. This is especially important since there are previous reports that both non-symptomatic and symptomatic smokers have elevated numbers of inflammatory cells and increased levels of cytokines such as interleukin-8 in their bronchial mucosa and sputum specimens [43,44]. There are, however, results also that stage 0 COPD does not necessarily lead to COPD progression. [21,22,45].

To conclude, our study reveals that several markers of oxidative/nitrosative stress and oxidant enzymes such as MPO and iNOS can be detected rather similarly in the sputum of non-symptomatic smokers and those chronic symptomatic smokers with normal lung function parameters who are considered to be at risk of developing COPD.

## Competing interests

The author(s) declare that they have no competing interests.

## Authors' contributions

PR took part in the planning of the study, laboratory analysis, calculated the statistics, prepared the tables and figures and participated in the writing process. TH participated in the recruitment and interview of the subjects and their characterization. HI has participated in the recruitment and interview of the subjects and their characterization. AR has interviewed part of the subjects.

AS has been responsible for the lung function analysis and FENO measurements. MM has taken part in the planning of the study, laboratory work and prepared the illustrations. VK was the principal investigator, has planned the study, made the literature research and most of the writing.
